# Unlocking opportunities to transform patient care: an expert insight on limitations and opportunities in patient monitoring

**DOI:** 10.1186/s40635-025-00733-z

**Published:** 2025-02-22

**Authors:** Maurizio Cecconi, Ana L. Hutanu, John Beard, Patricio Gonzalez-Pizarro, Marlies Ostermann, Anna Batchelor, Jos M. Latour, Jörn Grensemann, Michele Giovanni Mondino, Jesus Caballero, Manfred Blobner, Finn M. Radtke

**Affiliations:** 1 Humanitas University, Milan, Italy; 2 IRCCS Humanitas Research Hospital, Milan, Italy; 3 MedComms Consultant, Independent, UK; 4 GE HealthCare, Chicago, USA; 5 Department of Pediatric Anesthesia and Critical Care, La Paz University Hospital, Madrid, Spain; 6 IDIPaz Research Institute, Madrid, Spain; 7 Guy’s & St Thomas’ Hospital, London, UK; 8 The Newcastle Upon Tyne Hospitals NHS Foundation Trust, Newcastle Upon Tyne, UK; 9 School of Nursing and Midwifery, Faculty of Health, University of Plymouth, Plymouth, UK; 10 University Medical Center Hamburg-Eppendorf, Hamburg, Germany; 11 ASST Grande Ospedale Metropolitano Niguarda Ca’ Granda, Milan, Italy; 12 Intensive Care Medicine Department, Hospital Universitari Arnau de Vilanova Lleida, IRB Lleida, Spain; 13 School of Medicine and Health, Department of Anaesthesiology and Intensive Care Medicine, Technical University of Munich, Munich, Germany; 14 Faculty of Medicine, Department of Anaesthesiology and Intensive Care Medicine, Ulm University, Ulm, Germany; 15 Zealand University Hospital, Nykøbing F, Denmark; 16 University of Southern Denmark, Odense, Denmark

## Abstract

**Background:**

Current patient monitoring technologies are crucial for delivering personalised and timely care and are critical in achieving the best health outcomes while maintaining high care standards. However, these technologies also present several challenges affecting patients and healthcare professionals.

**Information overload:**

Healthcare providers often deal with excess data, making it challenging to identify the most critical patient information quickly. This may lead to delays in necessary interventions and potentially poorer patient outcomes.

**Alarm fatigue:**

Many patient monitoring systems trigger frequent false alarms. This high incidence can cause healthcare providers to become desensitised, potentially leading to slower response times or overlooked important alerts.

**Integration challenges:**

Current systems often need more seamless integration with other healthcare technologies, making it difficult for healthcare providers to have a cohesive view of the patient’s health. This lack of integration can impair care coordination and increase workloads. This paper presents the findings from a group of experts who described the state of the art of patient monitoring and discussed potential solutions and new pathways for developing these technologies.

## Introduction

Patient monitoring is crucial in ensuring patient safety, identifying clinical trajectories and deterioration, and allowing timely, efficient, and personalised interventions in healthcare. However, current monitoring technologies can be inaccurate, fail to maintain data continuity, or fail to promptly identify therapy response or patient deterioration [1, 2]. These limitations may lead to patient harm and overwhelm healthcare professionals (HCPs) with unnecessary or false alerts, leading to alarm fatigue and burnout [3], especially during staff shortages [4]. Many monitoring systems are immobile, hence non-transferable across settings, such as between intensive care units (ICU), operating rooms, and clinical wards. Current digital monitoring solutions can detect abnormal changes in the measured parameters, yet they do not identify underlying causes or assess individual organ function and can be inaccurate. For instance, the oxygen saturation sensor’s output accuracy is impacted by skin tone and colour, underserving patients from different racial groups [5]. Wearable devices can be uncomfortable, especially at night, highlighting the need for comfortable mobile wearables with high validity and precision [6].

This manuscript presents the key outcomes from the EMEA Patient Monitoring Advisory Board Meeting, held in June 2023 over 2 days in Glasgow, UK. The event was co-chaired by Maurizio Cecconi and John Beard. Participants were selected based on their expertise in patient monitoring and acute care, as well as their contributions to research and relevant publications. The Advisory Board comprised a mix of internationally renowned researchers and local experts, fostering diverse perspectives and balanced discussions. Following the meeting, participants continued their collaboration through email exchanges, culminating in the approval of this final draft.

The Board discussed unmet technological needs, exchanged insights, and shared experiences to raise awareness and initiate vital discussions within the healthcare and scientific community around the technological, biological, safety, sustainability, flexibility, tailored care, and psychological perspectives of patient monitoring. The experts have addressed specific questions and themes (Table 1) and have provided potential solutions that best serve patients, HCPs, healthcare systems, and the environment (Table 2).

**Table 1:** Key themes and questions for the future of patient monitoring

Clinical parameters	Care pathways
• Which physiological parameters are or not currently monitored? • What are the visual requirements of single and multiple parameters? • What are the current unmet technological needs?	• How can monitoring adapt between individual units? • How can monitoring span multiple units? • How can technology aid clinicians in implementing care pathways? • What are the current unmet technological needs?
Continuous physiologic monitoring in the medical-surgical unit	Novel technology implementation
• What proof is required establish the value of new wearable technologies apart from implementation and change management? • Which parameters could maximise value and improve outcomes in patient monitoring? • What is the preferred alarm data distribution method (technician reviews, bedside provider distribution, central station display, or escalation sequence to providers?	• What are the technology/resource unmet needs in supporting staff and deploying patient monitoring in clinical practice? • How can new wearable technologies be successfully deployed? • What are typical barriers to technology deployment? • Can technology enhance productivity and job satisfaction while reducing staff attrition/stress/burnout?
Patient monitoring and healthcare digital transformation	Remote patient monitoring and care at home
• What does digital transformation mean to you? • Which healthcare sectors could digital transformation positively affect, in your experience? • How can digital transformation influence patient monitoring- related activities? • What are your expectations from applying AI in these medical fields?	• Patients living with which conditions may benefit most from remote patient monitoring? • Is the clinical community ready to implement these technologies? • What are the technology gaps in remote monitoring? • What are the requirements for patient, their families, and the remote patient monitoring technology? • What is the status of the resulting cost burden?
The future of patient monitoring	
• What are the data access, integration, and infrastructure factors that must be considered? • What is the value of the ‘black box’ concept in patient monitoring?	• How can improvements on existing technologies facilitate practical implementation of future patient monitoring techniques?

**Table 2:** Potential solutions facilitated by technological advances in patient monitoring

Enhancing patient monitoring through thoughtful clinical parameters selection	Intelligent systems to reduce HCP workload and prevent burnout
• Identify and address gaps on organ-specific parameters, such as renal perfusion, brain function, and fluid management, to ensure comprehensive monitoring tailored to individual patient needs • Implement dynamic and integrated data presentation methods that allow for real-time monitoring and interpretation pf patient data (customisable visual reports and trend analysis tools to facilitate timely clinical decision-making)	• Utilise technology to streamline clinical workflows and reduce administrative burden on healthcare professionals (automated data visualisation tools, intelligent alerting systems, and machine algorithms to prioritise and contextualise patient data, enabling more efficient clinical decision-making) • Implement context-aware alarms that adjust alarm thresholds and priorities based on patient context, clinical urgency, and individual patient preferences to minimise alarm fatigue enhance patient safety
Patient-centred monitoring to support evidence-based clinical decision-making	Infrastructure and support facilitate healthcare digitalisation
• Consider individual patient needs, preferences, and circumstances when selecting monitoring approaches. This may include factors such as technical capabilities, home infrastructure, and personal support networks • Utilise wearable biosensors and remote monitoring technologies to provide continuous monitoring and early intervention, enabling proactive management of patient health outside of traditional healthcare settings	• Ensure reliable network connectivity by investing robust network infrastructure and leveraging technologies such as 5G and satellite communication of support remote monitoring and data transmission • Implement centralised data collection and management systems that aggregate monitoring data from various sources into a cohesive patient record. This centralised approach enables comprehensive patient monitoring, data-driven decision-making, and seamless information exchange between healthcare providers and departments
Seamless monitoring throughout care pathways to improve patient outcomes	Developing the future of patient monitoring
• Embrace digital transformation initiatives to establish intelligent infrastructure for end-to-end patient management. This involves implements interoperable systems that facilitate seamless data exchange different care settings, enabling comprehensive monitoring and timely intervention • Ensure data continuity by adopting universal monitor ports and continuous monitoring technologies that provide real-time insights into disease progression and response to patient location or care setting	• Foster collaboration between healthcare professionals, technology developers, researchers, and regulatory bodies to drive innovation in patient monitoring technology. This includes multidisciplinary research initiatives, industry partnerships, and regulatory frameworks that support developing and adopting novel monitoring solutions • Continue to advance the capabilities of patient monitoring technology by investing in research and development efforts focused on emerging technologies such as wearable sensors, artificial intelligence, and remote monitoring platforms. By staying at the forefront or technological innovation, healthcare organisations can ensure that patient monitoring remains effective, adaptable, and aligned with evolving healthcare needs

Following the meeting, the participants proposed collaborating on a paper outlining the future of patient monitoring. The lead authors prepared the initial draft based on their notes and discussions from the sessions. The draft was subsequently shared with all other authors, who contributed through email exchanges and revisions to refine and expand the content collaboratively.

The manuscript highlights the need for enhanced patient monitoring technologies that extend beyond intensive care units (ICUs), supporting broader patient populations across various hospital settings and ambulatory care. It advocates seamless integration of monitoring solutions across care pathways, guided by clinical specialists, to enhance patient outcomes, ensure data continuity, and reduce clinician workload. This holistic approach strives to improve patient safety, streamline clinical decision-making, and reduce the burden on healthcare providers by offering scalable, efficient, and patient-centred monitoring solutions throughout the entire healthcare journey.

### Enhancing patient monitoring through thoughtful clinical parameter selection

A current monitoring technology gap constitutes the lack of readily available organ-specific parameters crucial for decision-making and identifying factors conducive to recovery in complex patients [7]. Ideally, a monitoring system should be able to monitor renal perfusion, fluid management for acute circulatory dysfunction [8, 9], and monitoring of brain function in unconscious or sedated patients [10, 11]. Traditional stationary vital monitoring on wards restricts patients’ mobility, potentially delaying recovery [12]. Early detection of postoperative severe complications, such as bleeding, hypoxia, delirium, and sepsis [13], can be achieved through continuous monitoring of pulse rate, blood pressure, SpO_2_, temperature, and consciousness while ensuring patient mobility [14].

Artificial intelligence methods may help automate data visualisation and technology-assisted decision-making. Situation-awareness data visualisation tools and dynamic and integrated presentation would help clinicians digest information effectively, aid critical decision-making, and prevent burnout. For instance, visual outputs focused on clinical value trends rather than on absolute values would maximise the benefits of continuous monitoring [15]. Integrating multiple parameter trends and patterns could facilitate the differential diagnosis of underlying clinical conditions [16] where the clinical observation, such as hypotension, has numerous underlying causes. Various HCPs require different information to perform their roles, attuned to their level of training; therefore, visual reports should be adjustable to the user’s circumstances [17]. Developing alarm algorithms that cross-analyse trends across multiple parameters and facilitate clinical evaluation can enhance the accuracy of positive and negative alarm predictions.

### Patient-centred monitoring to support clinical decision-making

Patient selection is critical to patient-centred monitoring. Personalised requirements should be considered when selecting monitoring approaches, and wearable devices should facilitate this [18–20]. The intended monitoring length of time, the home or care setting infrastructure, and the support network are all considerations that emphasise the importance of social determinants of health under effective technology deployment. Personalised, holistic decision-making support based on precise parameters would provide early insights into a patient’s health trajectory.

High-quality at-home sensors can provide clinicians with actionable data before a patient reaches the hospital, enabling rapid access to clinical support. Such wearable technologies must be easy to operate, adjustable, reliable, reusable, accurate, and have extended battery life. Practical considerations include the need for devices to accommodate the diversity of human anatomy and day-to-day factors, such as monitoring patches that stay on when patients shower. New technologies and monitoring equipment should facilitate acute care treatment at home, pre- and post-hospitalisation, and during transit.

Technology offers the opportunity for novel patient monitoring approaches beyond conventional vital signs. For instance, accelerometers that detect patient movements may be precursors to fall events [21]. Similarly, non-contact camera-based technologies aim to evaluate parameters like respiratory rate [22, 23]. Smartphones are a portable device, potentially playing a central role in patient monitoring and management. Through downloadable apps, smartphones can track vital signs, medication adherence, and provide real-time health data, enabling remote patient monitoring. Despite challenges like data security and privacy concerns, smartphones remain essential in the ongoing transformation of healthcare to a more personalised, efficient, and accessible model [24]. On the other hand, point-of-care technologies (POCTs) encompass a broad array of tools that facilitate diagnostic, monitoring, and therapeutic interventions outside traditional clinical settings. These technologies often enable point-of-care testing, which focuses specifically on diagnostics. For instance, the widespread adoption of at-home COVID-19 swabs exemplifies the power of point-of-care testing to deliver rapid results without laboratory access. Emerging POCTs aim to monitor biomarkers in real-time, enabling continuous patient care and decision-making directly at the site of need and facilitating real-time results to be integrated into the patient’s electronic medical record (EMR) [25].

As disposable and wearable sensors proliferate, their utility should be balanced with environmental sustainability and clinical efficacy [26]. Wearable biosensors represent a subset of wearable devices that detect specific biological analytes, such as metabolites, hormones, and proteins in biofluids like sweat, saliva, and interstitial fluid. Recent advancements in wearable biosensor technologies include the development of multiplexed sensing approaches, microfluidic systems, and flexible materials for enhanced wearability, enabling precise and real-time physiological monitoring [19].

Sustainability is a pressing concern regarding the environmental impact of monitoring devices and their associated consumables and disposal process, as well as the economic sustainability and long-term financial considerations of implementing innovative digital solutions.

Remote monitoring for prehabilitation and postoperative recovery could enhance patient outcomes and resource usage. The value of such a tactic could be demonstrated by studying whether patient monitoring could reduce admission rates and inpatient time or potentially avoid surgery altogether. Remote monitoring should collect patient activity data, enabling a more precise assessment of cardiovascular conditions before physiologically stressful interventions [27].

### Seamless monitoring throughout care pathways to improve patient outcomes

Digital transformation will offer clinicians intelligent infrastructure to support end-to-end patient management for timely diagnosis, response to clinical interventions, prevention of adverse events, and recovery.

Data continuity along the care pathway represents a significant unmet need with consequences for patients and HCPs, particularly in critically ill patients who must be transferred. Implementing a single monitoring technology across all hospitals or hospital units is challenging. A practical solution could involve standardising connectivity via monitors with universal ports compatible with the sensors required in different settings, and communications standards enabling technology interoperability [26]. Compatibility between systems and devices is an additional challenge, which could be a requirement of procurement tenders when negotiating with technology companies. Future devices should use open, standardised protocols to enable data exchange independent of individual manufacturers’ systems. This innovation would ensure compatibility with various existing sensors and facilitate early recognition of deteriorating patients, allowing prompt intervention. This approach could optimise resource use and promote cooperation between HCPs and departments. Continuous, remote monitoring may prove superior to intrahospital live, intermittent vital sign checking, with initial studies revealing potential benefits in patient management and resource utilisation [7, 28–30]. However, more studies in broader patient populations are needed.

It is often critical to rapidly initiate monitoring and remote data access. Devices that can be used in different settings, such as ambulances and hospitals, would facilitate the workflow [20]. Clear roles and responsibilities regarding data management and action points would increase productivity and improve patient outcomes while reducing the unreasonable workload associated with staff burnout. Real-time monitoring and data-sharing between care teams may improve patient support in complex circumstances by connecting experts to frontline clinicians. This is similar to critical care telemedicine programmes that support remote or low-resource environments with virtual input from experts worldwide [31].

### Intelligent systems to reduce HCP workload and prevent burnout

Technology should reduce HCPs’ cognitive load and workload by streamlining and standardising administrative tasks to decrease bureaucracy. Studies assessing the impact of new technologies should include qualitative input from patients and HCPs while quantitatively analysing system efficiency. New monitoring technologies should undergo comprehensive human factors testing during development to maximise usability and minimise preventable errors [32]. Additionally, virtual or augmented reality technologies must be fully explored, tested, and understood before implementation [33].

Current efforts focus on integrating crew resource management (CRM) principles into healthcare, particularly in high-pressure settings like the ICU, to enhance patient safety by reducing human errors [34]. In a study, CRM strategies showed that high-fidelity simulation and education improved nurses’ communication knowledge and perceptions. Teams utilising these tools during simulations were notably more effective in problem identification, initiating critical interventions, and achieving positive outcomes, underscoring CRM’s potential to enhance interprofessional collaboration [34].

Access to real-time data allows monitoring of complex cases away from the ICU setting [35]. Central to this is tracking a patient’s progress or predicting deterioration, preferably before overt symptoms show. Early alerts targeting specific HCPs would allow clinical teams to distribute their attention efficiently. Physiologic scoring systems should be supported by timely data updates through continuous monitoring, facilitating multiple variable integrations to identify patients requiring resources. Appropriate at-home or on-ward monitoring could reduce the need for hospitalisation or transfer to the ICU, optimising resource utilisation while lowering overall costs.

Alarm fatigue for HCPs, particularly given the risk of false alarms, is a concern given the increase in monitoring, while frequent alarms are distressing to patients and their families [36]. Context-aware alarms should be developed with different protocols for specific scenarios, such as sleeping or active patients. Alarms could be removed from the bedside and redirected to specific clinicians; a need highlighted during the COVID-19 pandemic [37]. The outcome-based post-alarm analysis could optimise systems over time, decrease the risk of false alarms, and reduce ‘context noise’. Many ICUs still do not measure alarm fatigue despite the availability of validated questionnaires, which hampers efforts to address the issue. [38]. Tools such as the Charité Alarm Fatigue Questionnaire (CAFQa) can measure alarm fatigue in both nurses and physicians with nine items across two scales: ‘alarm stress’ and ‘alarm coping’ [39]. Benchmarking alarm fatigue in ICUs using these standardised instruments could help understand its burden and guide the evaluation of both technical and non-technical solutions to improve alarm culture and reduce alarm overload [39].

Artificial intelligence (AI) and machine learning may help automate data visualisation and technology-assisted decision-making. Computational techniques have been used to develop machine learning models to predict patient deterioration in real time based on standard features, such as demographics and laboratory data [40, 41]; yet further studies are required. Error filtering is crucial, and AI and machine learning could streamline data presentation while reducing the risk of storing erroneous data.

### Infrastructure and support to facilitate healthcare digitalisation

A robust network connection is essential for inpatient and at-home monitoring. Hospital zones with inadequate wireless connectivity should be identified and corrected. Remote monitoring appeals to patients who live in secluded areas and struggle with care access [42], although network coverage is a concern in these cases.

Complete digital transformation has broad requirements for HCPs, including equipping hospitals with information technology (IT) departments and infrastructure.

Centralised data collection from monitoring devices would allow cohesive monitoring to feed into complete patient records available at the bedside in an electronic format. Data should, therefore, be accessed through secured systems rather than on individual devices. Data security is paramount, and exposure risks or information leaks must be prevented at all costs to ensure confidence from patients and HCPs via transparent governance and ownership. Hospitals will need enclosed and secure networks for data access and storage, and information transfer should be seamless and accessible with minimal latency.

Updating healthcare IT systems is complex, and there are significant differences between countries regarding the pace of digitalisation, and local infrastructures must be prepared. Upfront costs may be considerable; yet, data showing the financial benefits of improved patient monitoring would demonstrate the financial value to healthcare systems and health insurance companies in a capital-controlled environment. For instance, hospital rapid response systems (RRS) rely on early tracking and recognition of patient deterioration. A meta-analysis of 29 studies revealed that RRS implementation was associated with reduced cardiopulmonary arrest and hospital mortality [43], and long-term costs. The IT departments must facilitate implementing and maintaining novel technologies, and all staff members should receive integrated, continuous training.

The digitalisation of healthcare also presents an opportunity to better identify patients who may qualify for certain important research studies early on; it also enables the integration of benchmarking to enhance the standard of care.

Another challenge in implementing patient monitoring technologies in ICUs and beyond is the lack of systematic application of existing methodologies, such as the Consolidated Framework for Implementation Research (CFIR) or the Non-Adoption, Abandonment, Scale-up, Spread, and Sustainability (NASSS) framework [44, 45]. These frameworks offer structured approaches to understanding the complexities of technology implementation, focusing on factors such as organisational culture, technology adaptability, and the readiness of healthcare systems [44]. However, they are not always utilised in the context of patient monitoring. The absence of these methodologies may result in fragmented or inefficient implementation strategies, where critical aspects like user acceptance, integration with existing workflows, and long-term sustainability are overlooked. By incorporating CFIR or NASSS, healthcare organisations could better assess barriers to adoption, identify the necessary resources for successful implementation, and develop tailored strategies to ensure patient monitoring technologies are both effectively integrated and widely accepted across different settings [44, 45].

Practical methods to bridge the gap between clinicians and manufacturers are needed to increase effective collaboration. One example of collaboration with potential applicability in patient monitoring is the use of healthcare hackathons. These events foster interdisciplinary collaboration by bringing together clinicians, patients, developers, and other stakeholders to co-create innovative solutions. Hackathons ensure clinicians are directly involved in the ideation and development stages, addressing real-world healthcare needs and improving user-centred outcomes. The approach has proven to be time-efficient, cost-effective, and more sustainable than traditional development models, allowing for rapid prototyping and feedback loops. By integrating clinicians early in the design process, hackathons can accelerate the creation of patient monitoring technologies that are both clinically relevant and user-friendly [46].

### Developing the future of patient monitoring

Enhanced patient monitoring technological solutions would improve clinical decision-making, reduce long-term costs, and increase efficiency, benefitting patients, HCPs, healthcare systems, and the environment. Scientists, biotechnology leaders, and HCPs of all specialities should engage with and become aware of the pressing issues that technology can address.

The experts concluded that there are several ways industry partners can help address these gaps, also taking on the responsibility of the development process. First, through collaboration, they can work closely with HCPs to understand real-world challenges and design devices that meet practical needs. Inclusivity is also crucial, ensuring devices are accurate across diverse patient populations and addressing issues like skin tone bias in sensors. Sustainability should be prioritised by developing reusable, eco-friendly devices and reducing the environmental impact of production and disposal. Interoperability is key, with a focus on creating devices that seamlessly integrate with existing hospital IT systems for better data-sharing and real-time access. Additionally, industry partners can support remote monitoring by creating reliable, easy-to-use home monitoring devices with secure data transmission and long battery life. Investment in AI and automation will enhance data analysis, reduce clinician workload, and improve patient outcomes. Lastly, it is essential to ensure hospitals have the necessary infrastructure, technology, and support to implement digital monitoring solutions effectively.

Let us further explore and promote the benefits of adopting digital monitoring solutions developed with the patient’s needs in mind. Let us advocate for healthcare systems to invest in solutions for high-risk, frail populations, prioritise organ-specific function monitoring, provide feedback to manufacturers to improve the devices, conduct high-quality studies, and publish the findings to help advance the field.

It is time to ignite meaningful conversations and fuel ethical improvements across healthcare systems beyond the EMEA region to improve healthcare practices and policies and create an accessible, flexible, and sustainable future in patient monitoring.

### Take-home message

There is a pressing need to improve patient outcomes and reduce healthcare provider burnout with patient-centred, environmentally conscious, and physiologic monitoring solutions. This article highlights the opportunities and needs for improved support of clinical decision-making, end-organ specificity, resource optimisation, and a sustainable healthcare future through more precise, integrated monitoring systems.

## Data Availability

Not applicable.

## Abbreviations

AI: Artificial intelligence
CAFQa: Charité Alarm Fatigue Questionnaire
CFIR: Consolidated framework for implementation research
CRM: Crew resource management
EMEA: Europe, Middle East, and Africa
HCP: Healthcare professional
ICU: Intensive care unit
IT: Information technology
NASSS: Non-adoption, abandonment, scale-up, spread, and sustainability
POCT: Point-of-care technology
